#  Importance of Educating Teenagers on Appropriate Safety Gear for E-Scooters. Comment on “Follow-Up Investigation on the Promotional Practices of Electric Scooter Companies: Content Analysis of Posts on Instagram and Twitter”

**DOI:** 10.2196/18945

**Published:** 2020-10-27

**Authors:** Claire SooHoo, Jackson SooHoo

**Affiliations:** 1 Geffen Academy at UCLA Los Angeles, CA United States

**Keywords:** e-scooters, trauma, electric scooter, public safety, road safety, public health, safety equipment, teenagers

My brother and I are writing a letter in response to the paper “Follow-Up Investigation on the Promotional Practices of Electric Scooter Companies: Content Analysis of Posts on Instagram and Twitter” by Dormanesh et al [1].

The paper discusses the promotion of proper safety equipment for riding electric scooters (e-scooters) online. The authors found that the social media accounts of prominent e-scooter companies Bird and Tier Mobility rarely, if ever, included photos of people wearing equipment such as helmets, knee pads, or wrist guards. To expand on this subject, Dormanesh and colleagues [1] could consider how e-scooter companies’ practices should better communicate or call attention to safety guidelines. We are both teenagers in high school and the dangers of e-scooters are familiar to us. The use of e-scooters, especially provided by rideshare companies such as Bird or Lime, has grown increasingly common among our peers in recent years. Especially in dense metropolitan areas, e-scooters can be extremely convenient for quick transportation. However, this ease of access also presents a problem of safety. We have noticed that in using these e-scooters, many of our fellow teenagers often fail to follow proper safety conventions. We almost never see anyone riding on a scooter with a helmet or other safety gear. Furthermore, there is no designated pathway for motorized scooters, and thus it can become hazardous for pedestrians on the sidewalk or on the road.

The importance of educating teenagers on appropriate safety gear is supported by recent research done reporting e-scooter injuries in the pediatric population. A previous study of 990 patients who sustained craniofacial injuries due to motorized scooter use shows that almost 50% of the injuries were to children under the age of 18 years, including 33% who were under the age of 12 [2]. A study conducted in Copenhagen revealed that over 10% of the patients studied who received injuries from using e-scooters were under the age of 18 [3]. Lastly, a study conducted locally at the University of California, Los Angeles, showed that 4 out of the 73 patients studied who sustained operative orthopedic injuries as a result of e-scooter use were adolescents [4]. All of these studies suggest that children and adolescents around the world are ignoring safety restrictions and riding e-scooters despite being underage. Although there are some restrictions for using the service such as owning a driver’s license and being at least 18 years old, these rules are easy to bypass. For example, many of our friends use their parents’ licenses to ride scooters. Stricter enforcement and more awareness of these regulations are necessary to decrease the risk of injury.

E-scooter accidents are currently difficult to accurately track in injury databases. A recent paper examined e-scooters using the “hoverboard” category from the National Electronic Injury Surveillance System, which includes electrically powered skateboards [5]. The lack of more specific coding results in unreliable data on the incidence of e-scooter injuries. We have also noted a similar issue with ICD-10-CM (International Classification of Diseases, Tenth Revision, Clinical Modification) coding. We attempted to examine the incidence of scooter injuries using the Patient Discharge Database in California. As a result of the absence of coding for e-scooter accidents, we used a proxy code, V03.19, which identifies pedestrians on conveyances other than roller skates or skateboards [6]. The demographics of this code are more consistent with e-scooter injuries than the code for motorized mobility scooters (V00.83), which included an older and less ethnically diverse group of patients (Table 1). Despite this, the lack of a more specific code limits our ability to accurately track e-scooter injuries. The implementation of proper coding for documentation in medical records would be valuable to researchers, hospitals, and the public in understanding the true risks and public health impact of e-scooter injuries.

**Table 1:** Demographics for the electronic scooter rider proxy code V03.19 compared to motorized mobility scooter riders (V00.83).

Patient characteristic	Electronic scooter proxy (n=402)	Motorized mobility scooters (n=165)	*P* value
Age (years), mean	51	68	<.001
Race/ethnicity (% White)	53	82	<.001
Insurance type (% uninsured)	6	2	<.001
Sex (% male)	69	85	<.001

## Abbreviations

e-scooter: electric scooter
ICD-10-CM: International Classification of Diseases, Tenth Revision, Clinical Modification
